# Welcome to *Journal of Angiogenesis Research*

**DOI:** 10.1186/2040-2384-1-1

**Published:** 2009-09-21

**Authors:** Mark Slevin, Yihai Cao, Jan Kitajewski

**Affiliations:** 1 Section of Vascular Biology, School of Biology, Chemistry and Health Science, Manchester Metropolitan University, Chester Street, Manchester, M1 5GD, UK; 2 Department of Clinical Biomedicine, ICCC, St Pau Hospital, Barcelona, Spain; 3 Department of Vascular biology, Microbiology, Tumor and Cell Biology, Nobels väg 16, Karolinska Institute, 171 77, Stockholm, Sweden; 4 Department of Reproductive Sciences, Columbia University Medical Center, Columbia, New York, USA

## Abstract

Angiogenesis is the growth of new blood vessels and is a key process which occurs during both physiological and pathological disease processes. Knowledge of the mechanisms through which this process is initiated and maintained will have a significant impact on the treatment of these diseases. Pathological angiogenesis occurs in major diseases such as cancer, diabetic retinopathies, age-related macular degeneration and atherosclerosis. In other diseases such as stroke and myocardial infarction, insufficient or improper angiogenesis results in tissue loss and ultimately higher morbidity and mortality.

## Editorial

The concept of tumor angiogenesis and therapy was proposed by Dr. Judah Folkman, who published the first and probably one of the most cited articles in 1971 [1]. In that article, Folkman showed and discussed early evidence that solid tumors could not grow beyond several millimetres in diameter without blood vessels and they might be held at the nonvascularized dormant stage. He proposed that tumors produce soluble factors to stimulate vessel growth and inhibition of tumor angiogenesis might be a novel approach for cancer therapy. While this hypothesis sounds logical and reasonable now, Folkman experienced unusually unsympathetic criticisms from his colleagues at that time. It took him nearly 30 years to convince the scientific community that his hypothesis was the correct one. We are all very grateful for Dr. Folkman's persistence, which has opened one of the most exciting and fast-expanding research areas in biomedical research. Today, millions of patients suffering from cancer and non-malignant diseases receive anti-angiogenic therapy. For those who work in this field, we are extremely fortunate to see Dr. Folkman's extraordinary achievements that have changed not only our lives but millions of patients.

Over the last couple of decades scientists have identified the importance of angiogenesis in determining disease development in association with tissue remodelling. It is now established that therapeutic modulation of the "angiogenic switch", may be a key mechanism for the treatment of these diseases. For these reasons and due to the continual expansion of this area of biomedical research, we are proud to announce the launch of *Journal of Angiogenesis Research*.

*Journal of Angiogenesis Research *aims to publish articles from all areas of the 'broad spectrum' of vascular research and from the bench to the bed-side. All submitted articles will be screened initially by a member of the journal's Editorial Board and if found to be suitable will be sent out for peer-review to internationally recognised experts in the field of angiogenesis research. The journal aims for the review process to be rapid and once accepted, the manuscripts will be published online immediately in a provisional format. Once an article is accepted for publication, the publisher will levy an Article Processing Charge which is a flat rate fee used to cover the article handling costs. Since articles are published online, there are no restrictions on size and number of figures that can be included. Articles will be immediately submitted to public repositories and will be freely available to read or down-load to readers world-wide.

Accepted articles will be of the highest quality and can take the form of original research articles, reviews and commentaries (some commissioned) providing pertinent, cutting edge up-dates in a focussed area of research. These can be in any field of research related to angiogenesis, from identification of novel mechanisms and signalling factors associated with angiogenesis, in vitro and in vivo disease models, results from pre-clinical trials, novel imaging modalities and nano-biological approaches. Please see the journal website for a more complete list of topics [2].

*Journal of Angiogenesis Research *welcomes your submissions, and hopes you will visit the website often to stay up-to-date with the latest articles.

## References

[B1] Folkman J (1971). Tumor angiogenesis: therapeutic implications. N Engl J Med.

[B2] Journal of Angiogenesis.

